# Sex differences and psychological stress: responses to the COVID-19 pandemic in China

**DOI:** 10.1186/s12889-020-10085-w

**Published:** 2021-01-07

**Authors:** Shiyan Yan, Rui Xu, Terry D. Stratton, Voyko Kavcic, Dan Luo, Fengsu Hou, Fengying Bi, Rong Jiao, Kangxing Song, Yang Jiang

**Affiliations:** 1 School of Acupuncture-Moxibustion and Tuina, Beijing University of Chinese Medicine, Beijing, 100029 China; 2 Institute of Basic Research in Clinical Medicine, China Academy of Chinese Medical Sciences, Beijing, 100700 China; 3 Department of Behavioral Science, College of Medicine, University of Kentucky, Lexington, KY 40536 USA; 4 Institute of Gerontology, Wayne State University, Detroit, MI 48202 USA; 5 School of Public Health, Central South University, Changsha, 410078 Hunan China; 6 Shenzhen Kangning Hospital, Guangzhou, 518020 China; 7 The First Clinical College, Hainan Meidical University, Haikou, 570100 China; 8 The First Medical Center, Chinese PLA General Hospital, Beijing, 100853 China

**Keywords:** COVID-19, Psychological stress, Sex differences, Psychological resilience

## Abstract

**Background:**

About 83,000 COVID-19 patients were confirmed in China up to May 2020. Amid the well-documented threats to physical health, the effects of this public health crisis - and the varied efforts to contain its spread - have altered individuals’ “normal” daily functioning. These impacts on social, psychological, and emotional well-being remain relatively unexplored – in particular, the ways in which Chinese men and women experience and respond to potential behavioral stressors. Our study investigated sex differences in psychological stress, emotional reactions, and behavioral responses to COVID-19 and related threats among Chinese residents.

**Methods:**

In late February (2020), an anonymous online questionnaire was disseminated via WeChat, a popular social media platform in China. The cross-sectional study utilized a non-probabilistic “snowball” or convenience sampling of residents from various provinces and regions of China. Basic demographic characteristics (e.g., age and gender) – along with residential living arrangements and conditions – were measured along with psychological stress and emotional responses to the COVID-19 pandemic.

**Results:**

Three thousand eighty-eight questionnaires were returned: 1749 females (56.6%) and 1339 males (43.4%). The mean stress level,as measured by a visual analog scale, was 3.4 (SD = 2.4) - but differed significantly by sex. Besides sex, factors positively associated with stress included: age (< 45 years), employment (unsteady income, unemployed), risk of infection (exposureto COVID-19, completed medical observation), difficulties encountered (diseases, work/study, financial, mental), and related behaviors (higher desire for COVID-19 knowledge, more time concerning on the COVID-19 outbreak). “Protective” factors included frequent contact with colleagues, calmness of mood comparing with the pre-pandemic, and psychological resilience. Males and females also differed significantly in adapting to current living/working, conditions, responding to run a fever, and needing psychological support services.

**Conclusions:**

The self-reported stress of Chinese residents related to the COVID-19 pandemic was significantly related to sex, age, employment, resilience and coping styles. Future responses to such public health threats may wish to provide sex- and/or age-appropriate supports for psychological health and emotional well-being to those at greatest risk of experiencing stress.

**Supplementary Information:**

The online version contains supplementary material available at 10.1186/s12889-020-10085-w.

## Background

Novel coronavirus disease 2019 (COVID-19) is now classified by the World Health Organization (WHO) as a full-blown pandemic. In China, where roughly 83,000 cases were confirmed in May 2020, a series of strict control measures were adopted which altered individuals’"normal” daily functioning [1]. Publicly, lack of an effective therapy, extended viral latency, asymptomatic infection, and talk of “post-recovery” reinfections exacerbated an already tense and uncertain situation [2–4]. Meanwhile, efforts to contain the spread, such as city lockdowns, home quarantines, and suspension of public and private services, resulted in behavior changes threatening individuals’ social, psychological, and economic well-being [5].

As “ground zero” for the pandemic, the COVID-19 threat is feared to have exacted a particularly dramatic psychological toll on the Chinese population. Indeed, a Hong Kong survey conducted during the early phases of the pandemic indicated that 97% of respondents were worried about COVID-19 - and 99% were alert to the disease progression, associated anxiety, and perceived susceptibility and severity [6]. Similarly, medical workers reported increased stress, anxiety, and depressive symptoms associated with the emergence, spread, and potential lethality of the virus [7, 8].

The potential impact of stress on anxiety, depression, and other neuropsychiatric disorders is well-documented [9, 10] – often, following disasters, resulting in post-traumatic stress disorder (PTSD) and substance use disorders [11]. Moreover, since the same preventive measures meant to limit the spread of an infection can paradoxically also heighten risks due to increased social conflict, isolation, devaluation, rejection, and exclusion, such efforts may be somewhat counterproductive [12]. These effects of increased, sustained psychological stress on individuals who, for various reasons, may be differentially impacted should not be discounted.

Sex is one such factor for concern. Clear sex differences have been shown to exist in exposure to potentially traumatic events and subsequent PTSD [13], and other studies have found females to be more vulnerable to developing mental or physical problems in response to life stressors or potentially traumatic events [14–16]. While early research has suggested that female medical workers may experience or respond more negatively to COVID-19-related events [8], the impact on psychological stress affected has not been fully investigated. This study, then, explores the psychological health and well-being of the general population in China, as well as factors – notably, sex - which may moderate its negative impact.

## Methods

In February of 2020, we administered to the general Chinese population an anonymous, online questionnaire using WeChat, a popular social networking platform. The cross-sectional study utilized a non-random “convenience” or “snowball” sample designed to elicit maximum participation by consenting adults. The study protocol received the appropriate human subjects review and approval.

The survey instrument was designed by several co-authors with psychiatric and epidemiological backgrounds (DL, KS, SY) and, in addition to basic information (e.g., sex, age, marital status, living conditions), asked about individuals’ experiences (e.g., the current pandemic status of COVID-19 in your area, who do you contact more than 3 times a week, desire to acquire knowledge of COVID-19, how much time concerning on the outbreak, and difficulty encountered during the pandemic, etc.) of and reactions to the COVID-19 pandemic – including psychological stress, resilience, and the perceived need for psychological support services (see Appendix). Psychological stress was measured using a 10-cm visual analogue scale (VAS) anchored at each end with *“not stressful”* (0) and *“extremely stressful”* (10, 17]. The Chinese version of the Connor-Davidson Resilience Scale (CD-RISC) was used to assess psychological resilience using 10 items ranging from 0 (*“Not at All”*) to 4 (*“Extremely True”*). For all measures, higher scores indicated higher levels of the respective construct [17].

### Statistical analysis

All analyses were based on 3088 participants, and descriptive statistics included univariate frequenciesand proportions. Multiple linear regression was used to analyze the influence of various independent variables on self-reported psychological stress, including: sex, age (< 45, ≥ 45 years) [18, 19], education, occupation, pandemic intensity (provincial prevalence of infections on 03/01/20), existing health conditions (0, 1–2, > 2), local pandemic status (on the rise, at the peak, steady, uncertainty), respondent’s infection status (confirmed, suspected, etc.), household size, frequency of personal contact, COVID-19 related health information needs, and emotional/mood responses. Marital status was found to be highly associated with household size, and thus was excluded from the model.

To explore sex differences, separate stepwise regression models were specified for males and females, with independent t-tests used to assess differences in mean psychological stress and CD-RISC scores. Differences in other measures, such as adaption to current living/working status, coping strategies for running a fever, and the perceived need for psychological support services were analyzed using Chi-square tests.

The specified critical *p* value for all inferential analyses was < 0.05, and all analyses were conducted using Statistical Analysis Software 9.4.3 (SAS institute Inc., Cary, NC).

## Results

A total of 3088 questionnaires were returned from residents across 32 Chinese provinces. Respectively, females and males comprised 56.5 and 43.5% of respondents; the average age was 37.5 years (SD = 13.5). Almost 1 in 5 (18.5%) were employed as medical personnel – 7.0% of whom reported working on the “front line” of the pandemic. The characteristics of participants are shown in Table 1. Item descriptions are shown in Table 1 of Appendix. The mean self-reported psychological stress score was 3.4 (SD = 2.4). The Cronbach’s alpha of CD-RISC was 0.913. The average psychological resilience score was 28.6 (SD = 8.1) (Table 2).

**Table 1:** The demographic information of the respondents (*n* = 3088)

Variables	Frequency(n)	Proportions(%)
Gender		
Male	1339	43.4
Female	1749	56.6
Age (years) Mean, SD	37.5 (13.5)	
< 45	2091	67.8
≥ 45	997	32.2
Marital status		
Single	978	31.7
Married	2014	65.2
Divorced	70	2.3
Widowed	26	0.8
Education		
Senior high school/technical secondary school and below	635	20.6
Associate degree in college	551	17.8
Bachelor’s degree	1448	46.9
Master’s degree and upper	454	14.7
Employment		
Front-line medical personnel	216	7.0
Non-front-line medical personnel	354	11.5
Military	107	3.5
Farmer	97	3.1
Worker	199	6.4
Government and management	373	12.1
Scientist	96	3.1
Teacher	299	9.7
Clerical and business	92	3.0
Service	320	10.4
Student	582	18.9
Unemployed	98	3.2
Others	423	13.7
Disease self-reported		
Without disease	2440	79.0
Respiratory disease	72	2.3
Infectious disease	17	0.6
Cardia-cerebrovascular disease	199	6.4
Disease of digestive tract	181	5.9
Endocrine disease	93	3.0
Urinary system diseases	51	1.7
Malignancy, anemia and other blood diseases	29	0.9
Surgical illness	71	2.3
Mental disorder	10	0.3
Others	140	4.5
Number of diseases self-reported		
0	2374	76.9
1–2	694	22.5
≥ 3	19	0.6

**Table 2:** Sex difference on psychology, behaviors and needs to cope with COVID-19

Variables	All respondents	Sex		
		Male	Female	*P*
Psychological stress, mean (SD)	3.4 (2.4)	3.6 (2.6)	4.0 (2.6)	0.0001
CD-RISC^a^, mean (SD)	28.6 (8.1)	29.6 (8.5)	27.8 (7.7)	< 0.0001
Adaption to current status n(%)				
Very adaptable	260 (8.4%)	106 (7.9%)	154 (8.8%)	0.0029
Able to adaptable	1503 (48.7%)	627 (46.8%)	876 (50.1%)	
Tolerated inadaptable	746 (24.2%)	315 (23.5%)	431 (24.6%)	
Inadaptable at most time	579 (18.8%)	291 (21.7%)	288 (16.5%)	
Coping strategy for running a fever n(%)				
Indecision	66 (2.1%)	37 (2.8%)	30 (1.7%)	< 0.0001
Go to fever clinic immediately	1698 (55.0%)	783 (58.5%)	915 (52.3%)	
Observe symptoms at home	1205 (39.0%)	460 (34.4%)	745 (42.6%)	
Recover by yourself	52 (1.7%)	32 (2.4%)	20 (1.1%)	
Others	67 (2.2%)	27 (2.0%)	39 (2.2%)	
Psychological support service needed				
Not needed	503 (16.3%)	240 (17.9%)	263 (15.1%)	0.0487
Telephone hotline	53 (1.7%)	27 (2.0%)	26 (1.5%)	
On line counseling	175 (5.7%)	84 (6.3%)	91 (5.2%)	
Self-adjustment methods	1265 (41.0%)	528 (39.4%)	737 (42.2%)	
Self-protection and precaution methods	673 (21.8%)	287 (21.4%)	387 (22.1%)	
Assessment of mental state	351 (11.4%)	138 (10.3%)	213 (12.2%)	
Others	67 (2.2%)	35 (2.6%)	32 (1.8%)	
Total	3088	1339	1749	

Notes: ^a^*CD-RISC* Connor-Davidson Resilience Scale for abbreviation, assessing the psychological resilience

Results of the multiple regression analysis, using psychological stress as the dependent variable, found several statistically-significant predictors, including being: (1) female; (2) < 45 years old; (3) more highly educated; (4) a farmer/worker/clerical and business/service; (5) unemployed; and (6) in poorer health. Having close contact or completing a medical observation, and a desire for know more about COVID-19 were also positively associated with self-reported stress. Variables significantly *negatively* related to psychological stress included: (1) frequent contact with colleagues; (2) calmness of mood comparing with the pre-pandemic; and (3) psychological resilience (Table 3).

**Table 3:** Multiple linear regression for psychological stress and its influence factors

Variables	All respondents		Male		Female	
	Estimates	*P* values	Estimates	*P* values	Estimates	*P* values
Intercept	7.4	<.0001	6.9	<.0001	9.4	<.0001
sex	0.3	0.0036	–	–	–	–
Age(< 45, ≥45 years)	−0.4	<.0001	−0.4	0.0078	−0.4	0.0009
Education	0.2	<.0001	0.3	<.0001	–	–
Employment (Military as reference)						
Unsteady income^a^	0.4	0.0002	0.4	0.007	–	–
Unemployed	0.6	0.0211	–	–	–	–
Number of diseases self-reported	0.3	0.0041	–	–	0.4	0.0077
Local epidemic status	–	–	–	–	−0.3	0.0059
Risk level of the population ^b^	−0.9	0.0038	−1.2	0.0057	–	–
Contacted more than 3 times a week with colleagues	0.3	0.0013	–	–	0.4	0.0007
Desire to acquire knowledge of COVID-19	−0.2	0.0006	–	–	−0.3	<.0001
Time spent on the outbreak in one day	0.1	0.0396	0.2	0.007	–	–
Mood changes comparing with the early epidemic phase	−0.4	<.0001	−0.4	<.0001	−0.4	<.0001
Difficulties encountered (Social limitations as reference)						
Diseases problems	0.5	0.0235	–	–	0.8	0.0086
Psychological problems	1.2	<.0001	1.1	0.0002	1.3	<.0001
Economic problems	0.8	<.0001	0.8	0.0004	0.9	<.0001
Unable to work/study	0.3	0.0072	–	–	0.3	0.0357
CD-RISC	−0.1	<.0001	−0.1	<.0001	−0.1	<.0001

Notes: ^a^ Unsteady income were including farmer, worker, clerical, business and service; ^b^ Close contact or completed a medical observation vs. general people

### Male and female stressors

Except for age, high education, and resilience, for male, unstable income population (farmer/worker/clerical and business/service), more time concerning on the COVID-19 outbreak, risk infection (close contact or completed a medical observation), psychological and economic problems aggravated psychological stress. In female, poorer health, worse local pandemic status, higher desire for knowledge about the COVID-19, problem of diseases during the pandemic and unable to work/study were the risk factors. Calmness of mood comparing with the pre-pandemic and frequently contacting with colleagues were protective factors of stress (Table 3).

Sex differences did exist with regard to adaptation, responses to potential symptoms, and the perceived need for psychological support services – with males being less adaptive and less likely to see a need for psychological support, but more likely to seek immediate medical attention for suspected feverish symptoms (see Table 2).

## Discussion

Research has consistently shown that major life changes can lead to severe and sometimes chronic psychological stress [20]. With more than 2.1 million cases confirmed worldwide, and over 140,000 reported deaths at the time of this study [21], the COVID-19 pandemic constitutes a pervasive source of potential stress on a global scale. Indeed, with many countries swiftly instituting strict control measures, normal routines were drastically disrupted with the closing of businesses, industries, and schools – and the recommendation (or requirement) that individuals remain at home. Such behavioral changes, whether mandatory or not, can be expected to negatively impact individuals’ mental health and/or emotional well-being.

Our study, conducted across provincial China (Taiwan excepted) at the height of the COVID-19 pandemic, suggests that being female, somewhat younger (< 45 years old), more highly educated, unemployed, and in poorer overall health were all risk factors for experiencing psychological stress. Uncertainty of one’s local state pandemic status and some prior personal COVID-19-related contact were also factors contributing to one’s perceived stress. In contrast, “protective” factors included frequent contacting with colleagues, calmness of mood comparing with the pre-pandemic, and psychological resilience. A desire for knowledge about COVID-19 and being unable to go to work/study were additional risk factors for stress. The results consistent with similar COVID-19 worldwide studies [22–26].

Disease susceptibility, and the economic problems that can result from an inability to work, can be prime contributors to psychological stress [27, 28]. Similarly, uncertainty and lack of control resulting from lockdowns, restrictions, quarantines, etc. arguably impacted every Chinese residents’ life and, potentially, their physical, social-psychological, and economic well-being [2, 3]. The sustained, long-term implementation of these safeguards undoubtedly prolonged an already stressful and challenging situation – and the loss of both income and personal identity associated with the lack of employment likely resulted in increased anxiety. Indeed, for respondents without sustainable incomes, the effects were especially dire.

The full range of possible impacts should be considered when implementing disease control and prevention measures, and disseminating understandable disease-related information and providing alternative venues for personal contact with friends or colleagues couldbe effective buffers of psychological stress. Conforming to common perceptions, people alerted to COVID-related risks and threats instinctively seek outside help, confirmed in a recent Chinese study demonstrating that individuals, on average, spent ≥3 h per day during the pandemic associated with mental health [29]. Social support, typically associated with lower depression and anxiety, could further buffer the cognitive effects of stress [30]. Our findings suggest that appropriate social supports (e.g., frequent contactwith colleagues) to relieve stress during a pandemic might include providing more professional knowledge of protective measures, real-time updates and reports, access to urgent medical services, basic living security measures, and alternative means to interpersonal communication.

Age was another factor related to self-reported stress – with study findings suggesting that younger (< 45) respondents experienced greater stress. These results were also consistent with the previous COVID-19 studies of the psychological impacts of disasters [29, 31]. Exactly why this is the case remains somewhat unclear: Perhaps older persons direct more cognitive effort to maintaining positive emotions and avoiding negative ones(“positive effects”) [32]. Conversely, maybe younger residents face greater social, emotional, and/or economic responsibilities toward their families’ health and protection.

Our study also highlighted the importance of resilience as a “protective” factor to psychological stress, often vis-à-vis a greater sense of adaptability and control over one’s external environment. In fact, studies have found that psychological resilience both directly and indirectly protects some individuals against stress-related mental health problems (e.g., PTSD, anxiety, depression) [33, 34]. In our study, males’ higher resilience may partially explain their comparatively (vs. females) lower stress levels.

That female’s stress was greater than that of males was also consistent with existing evidence [14, 16] and similar studies conducted during the pandemic of COVID-19 from different countries [22–25]. The finding correspondents to epidimeological research suggesting that females have a higher risk of psychological outcomes [35]. Some researchers have hypothezied that higher psychological stress in females may be partially due to their work being more heavily impacted by COVID-19 and the care burden in home [22, 25]. Observed sex differences regarding stress are also often attributed to differential impacts on individuals’ social environmental, psychodynamic, and cognitive processes [36, 37]. Behavioral responses to distress and the experience/expression of emotion are also thought to be moderated by sex [13] and, more recently, sex differences in susceptibility to stress have been expanded to include physiological factors [38, 39] such as ovarian hormone fluctuations [36, 40] and endogenous estradiol changes across the menstrual cycle [41]. Similarly, stress-related fMRI studies have found brain function associated with emotion and stress regulation, self-referential processing, and cognitive control to be more pronounced in males [42]. Sex differences in self-reported stress are further reflected in the perceived need of psychological support services, which are often most evident in females. The sex differences of stress should be paid attention, and the government should provide appropriated paychological support services to improve female’s resilience and alleviate their distress [23].

This study had several limitations. First, although study respondents reflect a national sample, much of the non-random, convenience sample was located outside the heaviest pandemic area. Moreover, the cross-sectional design makes establishing the causal nature of relationships problematic. Second, in response to the then-rising COVID-19 pandemic, our use of the WeChat platform may have had some systematic effect on participation. Finally, for ethical reasons, we did not ask about confirmed or suspected infection among respondents; however, the proportion reporting close contact with an infected individual and/or having confirmed medical tests for the virus was quite small.

## Conclusion

Self-reported stress, as measured, was significantly related to sex, age, education, and employment during the pandemic of COVID-19. Findings suggest responses to future emergency situations (e.g., disease pandemics) should bolster appropriate control measures with targeted means of psychological support.

## Supplementary Information

**Additional file 1: Appendix Table 1**: Description of pandemic and living status characteristics related to COVID-19. Description of data: **Table 1** showed the pandemic and living status information of the participants during the COVID-19 pandemic. **Questionnaire**: The Questionnaire of the Public Psychological Status during the COVID-19 pandemic period. Description of data: It provided the questionnaire used in our study.

## Data Availability

The data and material can be accessed from Kangxing Song, skx301@126.com.

## Abbreviations

WHO: World health Organization
PTSD: Post-traumatic stress disorder
VAS: Visual analogue scale
CD-RISC: Connor-davidson resilience scale
