# Supplementary material for: Sex differences and psychological stress: responses to the COVID-19 pandemic in China
Source: BMC Public Health. 2021 Jan 7;21:79. doi: 10.1186/s12889-020-10085-w (PMC7789895; doi:10.1186/s12889-020-10085-w)
Supplement: Supplementary file 1 — Additional file 1: Appendix Table 1: Description of pandemic and living status characteristics related to COVID-19. Description of data: Table 1 showed the pandemic and living status information of the participants during the COVID-19 pandemic. Questionnaire: The Questionnaire of the Public Psychological Status during the COVID-19 pandemic period. Description of data: It provided the questionnaire used in our study. [file 12889_2020_10085_MOESM1_ESM.docx]

**Appendix**

[Table 1 Description of epidemic and living status characteristics related to COVID-19 2](#_Toc26976)

[The Public Psychological Status Questionnaire during the COVID-19 epidemic period 4](#_Toc20336)

# Table 1 Description of pandemic and living status characteristics related to COVID-19

| Variables | Frequency（n） | Proportions（%） |
| --- | --- | --- |
| Assessment of local pandemic status in your area |  |  |
| On the rise | 97 | 3.1 |
| At the peak | 43 | 1.4 |
| Steady | 2607 | 84.4 |
| Uncertainty | 341 | 11.0 |
| Which classify is appropriate to you |  |  |
| Confirmed patients | 0 | 0.0 |
| Suspected infection | 0 | 0.0 |
| Close contact | 21 | 0.7 |
| Person who has completed a medical observation | 41 | 1.3 |
| General population | 3026 | 98.0 |
| How many persons living together during the outbreak |  |  |
| Alone | 225 | 7.3 |
| 2-5 | 2638 | 85.4 |
| 6-10 | 163 | 5.3 |
| 11-20 | 18 | 0.6 |
| ＞20 | 44 | 1.4 |
| People contact more than 3 times a week besides those you live together |  |  |
| Family members | 2152 | 69.7 |
| Friends | 937 | 30.4 |
| Colleagues | 1083 | 35.1 |
| Classmates | 518 | 16.8 |
| Wardmates | 26 | 0.8 |
| Medical staff | 155 | 5.0 |
| Net friends | 138 | 4.5 |
| Others | 190 | 6.2 |
| Desire to acquire knowledge of COVID-19 |  |  |
| Great | 1036 | 33.6 |
| Relatively great | 1079 | 34.9 |
| General | 885 | 28.7 |
| Relatively small | 48 | 1.6 |
| Not at all | 40 | 1.3 |
| Time concernning on the outbreak in a day |  |  |
| ＜30minus | 1001 | 32.4 |
| 30-60minus | 1425 | 47.0 |
| 1-2hours | 349 | 11.3 |
| ＞2hours | 286 | 9.3 |
| What's your current mood comparing with the early pandemic phase |  |  |
| More depressed | 366 | 11.9 |
| Depressed | 450 | 14.6 |
| No change | 349 | 11.3 |
| Calmness | 1923 | 62.3 |
| The greatest difficulty encountered during the pandemic |  |  |
| Problem of diseases | 104 | 3.4 |
| Psychological problems | 194 | 6.3 |
| Economic problems | 273 | 8.8 |
| Inconvenience in daily life | 1360 | 44.0 |
| Unable to work/study | 732 | 23.7 |
| Social limitations | 425 | 13.8 |

**Questionnaire**

**The Questionnaire of the Public Psychological Status during the COVID-19 pandemic period**

Dear friend, the breakout of COVID-19 impacted all avenues of modern life. The following brief questionnaire asks you to voluntarily share your own experiences during this unprecedented time. Completion of the short instrument takes approximately 15 minutes and is completely anonymous; you cannot be linked to your responses. Won't you please take a few minutes and help us better understand the social and psychological reactions to this ongoing development? Completion of the short instrument takes approximately 10 minutes and is completely anonymous; you cannot be linked to your responses. Won't you please take a few minutes and help us better understand the social and psychological reactions to this ongoing development? Thank you for your participation!

1．What is your sex?

A．Male

B．Female

2．What is your age?

years old.

3．What is your marital status:

A．Single

B．Married

C．Divorced

D．Widowed

4．What is the highest level of school you have completed or the highest degree you have received?

A．Senior high school/technical secondary school and below

B．Associate degree in college

C．Bachelor's degree

D．Master's degree

E．Doctoral degree

5．Which of the following best indicates your occupation?【Multiple choices is allowed】

A．Front-line medical personnel

B．Non-front-line medical personnel

C．Soldier

D．Farmer

E．Worker

F．Government and management

G．Scientist

H．Teacher

I．Clerical and business

J．service

K．Student

L．unemployed

M．Others

6．In which province or area do you currently live?:（drop-down option）

7．Do you suffer from any of the following diseases（including past and present）【Multiple choices is allowed】：

A．Without disease

B．Respiratory diseases: pneumonia, asthma, branching, etc

C．Infectious diseases: SARS, tuberculosis, hepatitis b, etc

D．Cardiovascular and cerebrovascular diseases: hypertension, coronary heart disease, heart failure, cerebral infarction, cerebral hemorrhage, etc

E．Digestive tract diseases: gastritis, gastric ulcer, etc

F．Endocrine diseases: diabetes, hyperthyroidism, etc

G．Urinary diseases: kidney failure, stones, infection, etc

H．Malignancy (cancer), anemia and other blood diseases

I．Surgical illness (operated on or requiring surgery)

J．Mental disorder

K．Others

8．What's the current pandemic status of COVID-19 in your area?

A．On the rise (Cases are increasing rapidly)

B．At the peak(The growth of cases is at the peak)

C．steady (New cases are growing slowly or falling)

D．Uncertainty

9．Which classify is appropriate to you?

A．Confirmed patients

B．Suspected infection

C．Anyone in close contact

D．Person who has completed a medical observation

E．General people

10．How many people do you live together during the COVID-19 outbreak?

A．Alone

B．2-5 persons

C．6-10 persons

D．11-20 persons

E．>20 persons

11．Who do you contact more than 3 times a week besides those you live together【Multiple choices is allowed】：

A．Family members

B．Friends

C．Colleagues

D．Classmate

E．Ward-mates

F．Medical staff

G．Net friends

H．Others

12．To what degree your desire to acquire knowledge of COVID-19:

A．Great

B．Relatively great

C．General

D．Relatively small

E．None

13．How much time concerning on the outbreak in a day :

A．＜30 minutes

B．30-60 minutes

C．1-2 hours

D．＞2 hours

14．What's your current mood comparing with the early pandemic phase：

A．More depressed

B．Depressed

C．No change

D．Calm

15．The greatest difficulty you encountered during the pandemic:

A．Problems of diseases

B．Psychological problems

C．Economic problems

D．Inconvenience in daily life

E．Unable to work/study

F．Social limitations

16. Do you adapt to current living and working status:

A. Very adaptable

B. Able to adaptable

C. Tolerated inadaptable

D. Inadaptable at most time

17.If you find yourself running a fever, the first thing comes to your mind is:

A.Go to a fever clinic immediately

B.Observe symptoms at home

C.Recover by yourself

D.Indecision

E.Others

18．Over the last two weeks, how often have you been bothered by any of the following problems?

0=Not at all 1=Several days 2=More than half the days 3=Nearly everyday

（1）Little interest or pleasure in doing things?

（2）Feeling down, depressed, or hopeless?

（3）Feeling nervous, anxious or on edge?

（4）Not being able to stop or control worrying?

1. Over the past month, choose the statement that best fits your situation(Connor-Davidson Resilience Scale).

0=Not true at all 1=Rarely true 2=Sometimes true 3=Often true 4=True nearly all of the time

（1）Able to adapt to change

（2）Can deal with whatever comes

（3）Tries to see humorous side of problems

（4）Coping with stress can strengthen me

（5）Tend to bounce back after illness or hardship

（6）Can achieve goals despite obstacles

（7）Can stay focused under pressure

（8）Not easily discouraged by failure

（9）Thinks of self as strong person

（10）Can handle unpleasant feelings

20．To what degree you experienced psychological stress during the past month?

21．Which psychological services do you urgently need at present?

A．Not needed

B．Telephone hotline

C．On line counseling

D．Self-adjustment methods

E．Self-protection and precaution methods

F．Assessment of mental state

G. Others:
